# Saved by the Scan: A Case of Early Detection of Sarcomatoid Mesothelioma

**DOI:** 10.7759/cureus.26793

**Published:** 2022-07-12

**Authors:** Hasan Choudhury, Jason Budde, Nayab Ahmed, Andrew Johnson, Comfort Adewunmi

**Affiliations:** 1 Internal Medicine, Northeast Georgia Medical Center Gainesville, Gainesville, USA; 2 Cardiothoracic Surgery, Northeast Georgia Medical Center Gainesville, Gainesville, USA; 3 Oncology, Northeast Georgia Medical Center Gainesville, Gainesville, USA

**Keywords:** tobacco abuse, immuno-checkpoint inhibitor, immuno-chemotherapy, preventative care, localized malignant mesothelioma

## Abstract

We report a case of localized sarcomesothelioma detected during screening via a low-dose CT (LDCT) scan. The patient is a 71-year-old female, a current 56-pack-year cigarette smoker with a past medical history of myocardial infarction and stroke with a Zubrod score of zero. A screening LDCT revealed a 1.9 cm × 1.8 cm × 1.4 cm right lower lobe lesion with smooth margins and close association with the hemidiaphragm. A wedge resection with biopsy showed high-grade sarcomatoid mesothelioma with extensive desmoplastic morphology and negative margins. The patient opted for imaging surveillance, and at 12 months has shown no evidence of tumor recurrence on positron emission tomography (PET)/CT. The case shows that LDCT screening discovers cancers and saves lives. It also presented a dilemma for the patient and her oncologist because common guidelines do not define a recommended treatment.

## Introduction

Malignant pleural mesothelioma (MPM) is an aggressive tumor originating from mesothelial cells lining the lung pleura, peritoneum, tunica vaginalis, and pericardium, listed in descending order of occurrence [1]. The incidence of MPM in the United States in 2018 was 0.7 per 100,000 people [2]. The three main histological subtypes of MPM include epithelioid, sarcomatoid, and biphasic [1], with sarcomatoid being the least common, accounting for 10% of all cases, and the most aggressive type. There is also a subtype of sarcomatoid mesothelioma called desmoid sarcomesothelioma [1], which is the focus of this report. The occurrence of MPM is attributed to mineral fiber exposure such as asbestos, and the median duration of time to develop mesothelioma following asbestos exposure is 32 years [3-5]. MPM is most commonly diagnosed once symptoms manifest, or at least with major radiographic abnormalities. According to 2018 SEER data, almost three times as many patients presented with distant spread as compared with localized disease [2]. Patients have an average survival rate of seven to twelve months from the time of diagnosis of MPMs [4]. We report here a very unusual case where a patient’s localized, completely resectable desmoid sarcomesothelioma was detected on a screening low-dose CT (LDCT) scan.

## Case presentation

**Figure 1:**
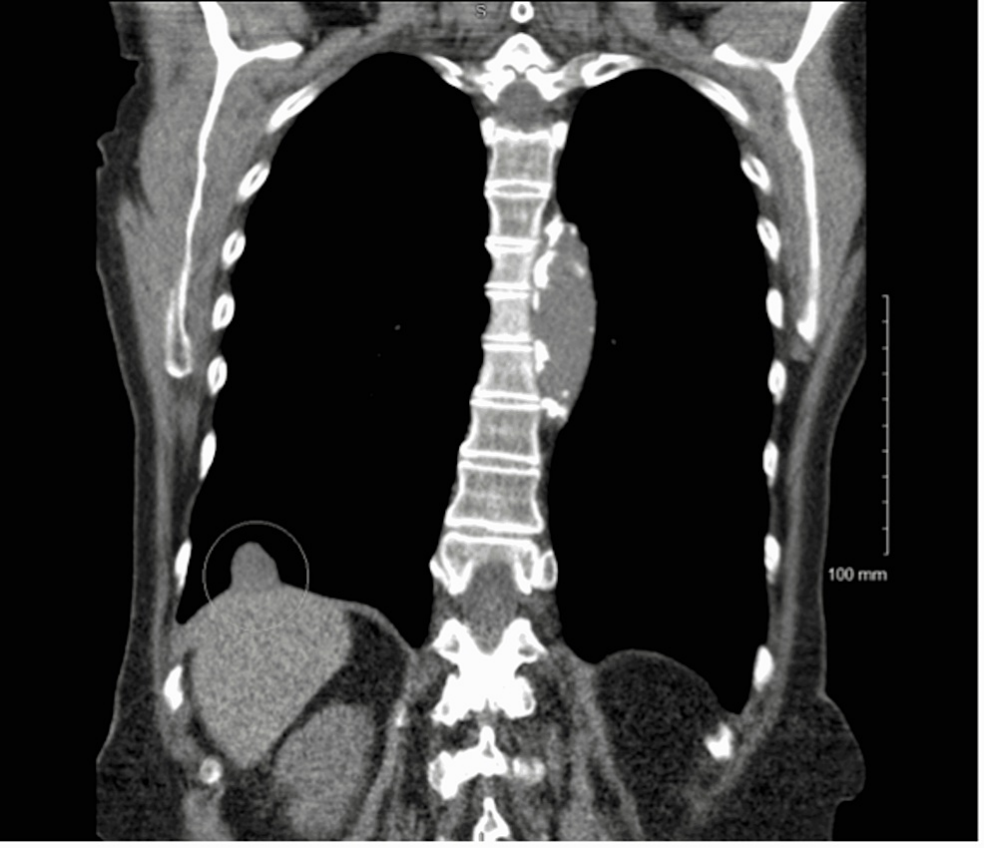
Low dose CT scan posteroanterior view Low dose CT scan PA view showing the 1.9 cm × 1.8 cm × 1.4 cm mass with smooth margins abutting the right hemidiaphragm

**Figure 2:**
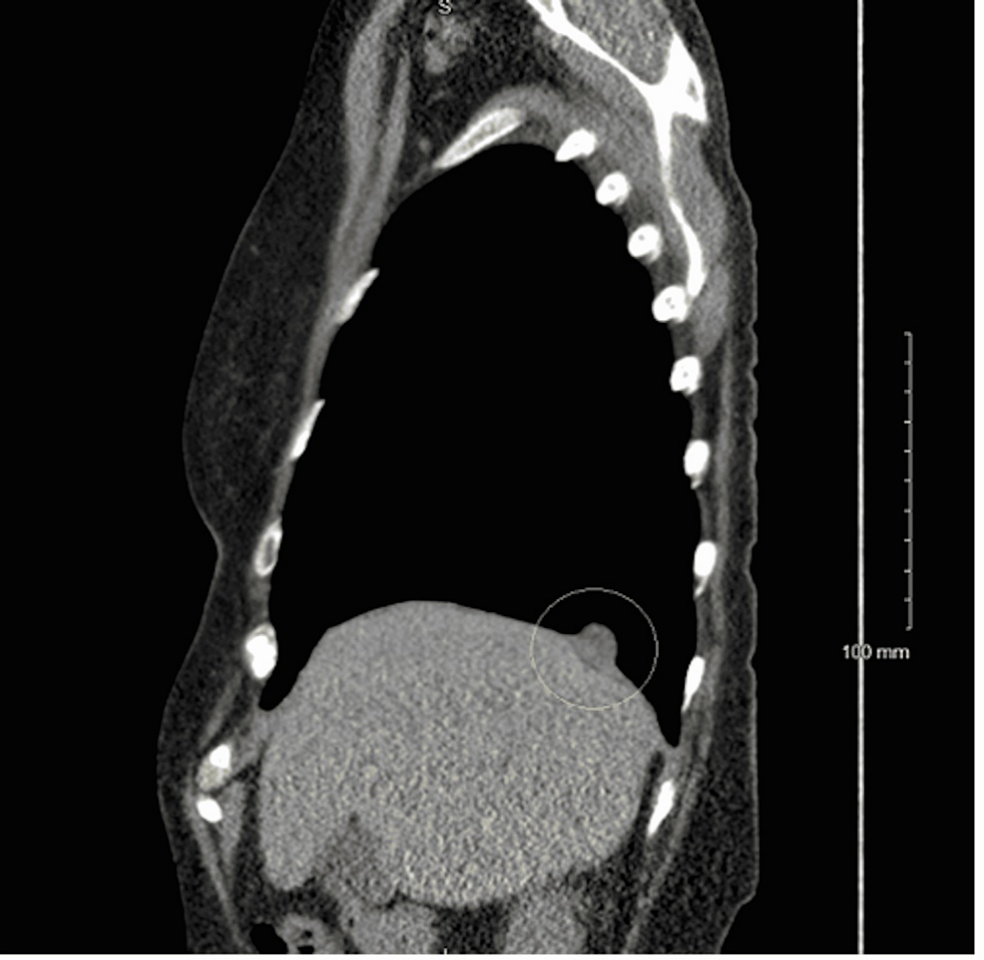
Low dose CT scan lateral view Low dose CT scan lateral view showing the 1.9 cm × 1.8 cm × 1.4 cm mass with smooth margins abutting the right hemidiaphragm

**Figure 3:**
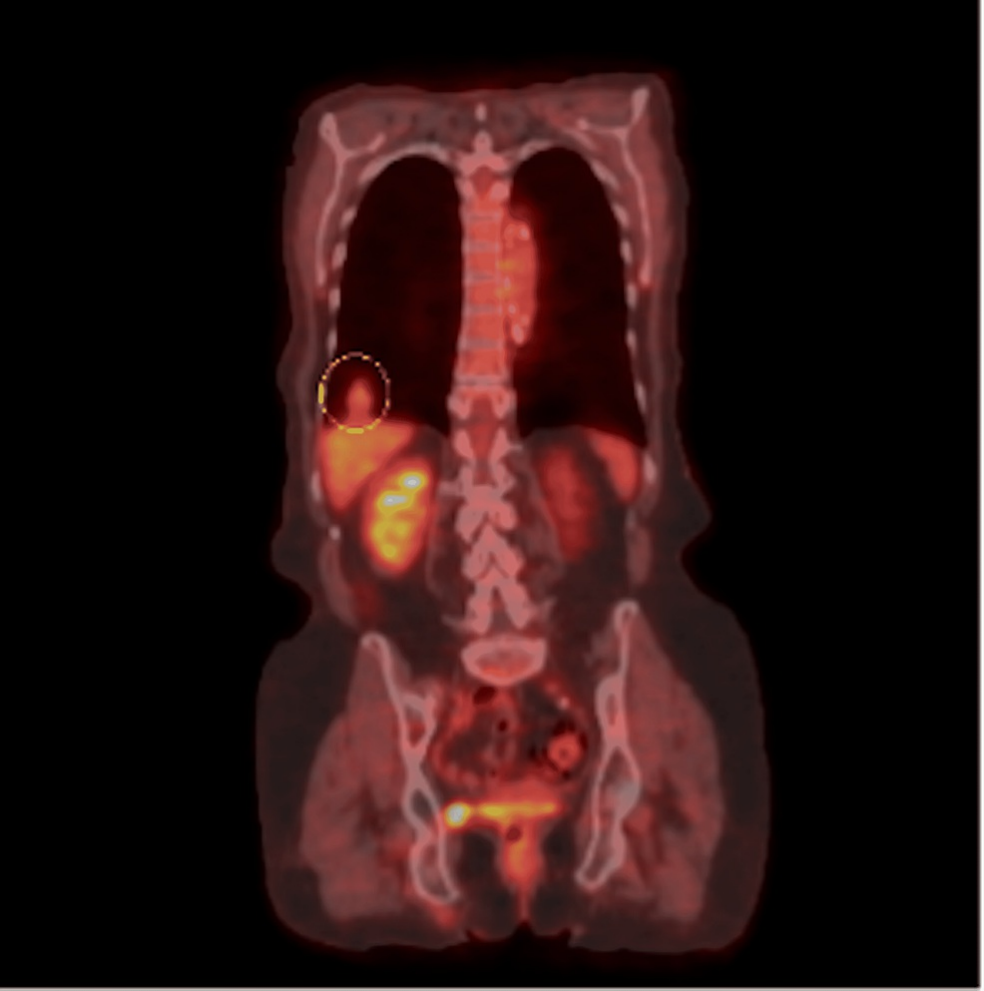
PET scan Hypermetabolic activity within the lesion with a maximum SUV of 2.6 without evidence of nodal involvement

The patient is a 71-year-old female, a current 56-pack-year cigarette smoker with a past medical history of myocardial infarction and stroke, with a Zubrod score of zero. She denied fevers, chills, night sweats, hemoptysis, or unexpected weight loss and did not have occupational exposure to asbestos. A screening LDCT revealed a 1.9 cm × 1.8 cm × 1.4 cm right lower lobe lesion with smooth margins and close association with the hemidiaphragm (Figures 1-2). A whole-body positron emission tomography (PET) scan (Figure 3) showed mild hypermetabolic activity within the lesion with a maximum standard uptake value (SUV) of 2.6 without evidence of nodal involvement. CT-guided biopsy was inconclusive, with findings of a small amount of fibrous scar-like tissue with chronic inflammation mixed with mesothelial lined tissue, alveolar lung parenchyma, and striated muscle.

Given the overall clinical history and imaging, it was decided that the patient required additional biopsy via either a repeat CT-guided needle or surgical excision. Because of the proximity to the diaphragm, a repeat needle biopsy was considered too difficult, and she was referred to a cardiothoracic surgeon.

**Figure 4:**
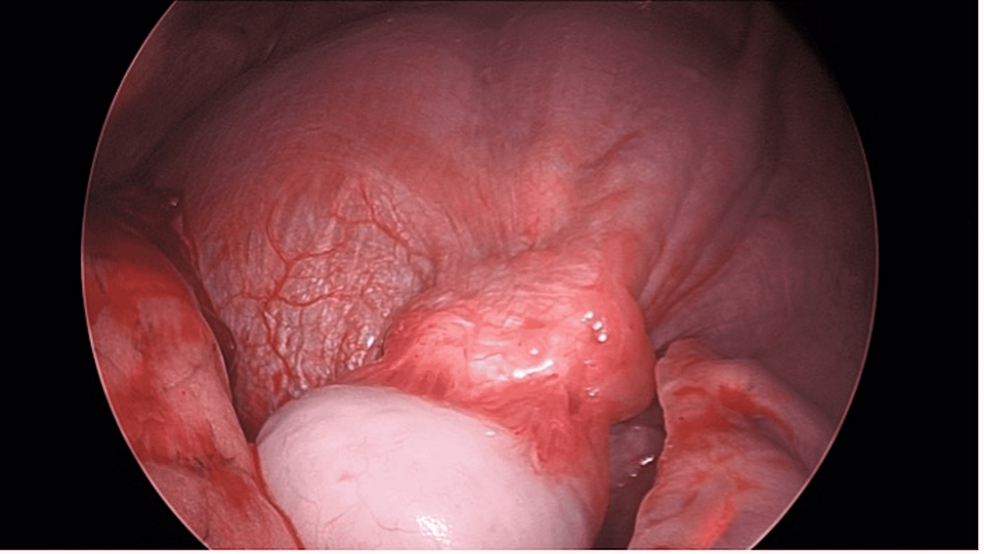
Mass prior to resection Image of the mass during video-assisted thoracoscopic surgery showing the mass prior to resection

**Figure 5:**
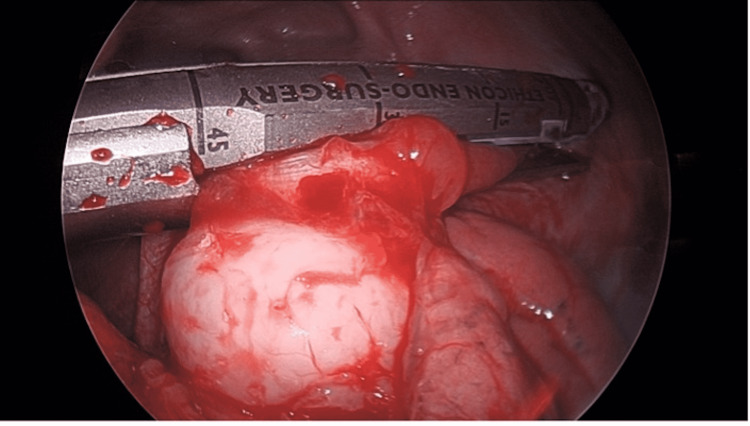
Image of mass after staple placement Image of the mass during video-assisted thoracoscopic surgery showing the mass with the staple in place

Right, video-assisted thoracoscopic surgical (VATS) wedge resection of the mass, incorporating small but adequate margins of lung and diaphragmatic muscle, was performed (Figures 4-5). Because lymph nodes were neither enlarged nor "hot" on the PET scan, they were not sampled. The intraoperative frozen section was able to confirm the presence of malignancy without further identification, which would require additional studies. The margins were negative for a tumor on a frozen section.

Final pathologic analysis revealed a 2 cm high-grade sarcomatoid mesothelioma with extensive desmoplastic morphology and negative margins. The immunostains were positive for calretinin, MCK, GATA3, patchy positivity for CK7 and pan keratin, and rare cells positive for WT-1.

The patient was referred to a medical oncologist; the patient opted for imaging surveillance, and at 12 months has shown no evidence of tumor recurrence on PET/CT.

## Discussion

The above case is notable because it shows that LDCT scans discover cancers and save lives. This is also demonstrated by National Lung Screening Trial data showing a 20% relative reduction in mortality from non-small cell lung cancer with LDCT compared to chest X-rays [6]. Second, this case highlights the novelty of small and resectable sarcomatoid mesotheliomas, which have been described only in individual case reports. Relatedly, the case presented a dilemma for the patient and her oncologist because standard guidelines, including the National Comprehensive Cancer Network (NCCN), do not define a recommended treatment due to the rarity of resectable sarcomatoid types [7]. While she was offered adjuvant ipilimumab and nivolumab, as approved by the United States Food and Drug Administration (FDA) as adjuvant therapy, she decided for ongoing screening [8]. A review of the literature also indicates that approaches such as chemotherapy with tumor treating fields (TTF) or pegylated arginine deaminase (ADI-PEG 20) are possible therapeutic approaches for mesothelioma [9,10]. Although it is difficult to estimate the patient’s expected prognosis, it is documented that the five-year survival rate of localized malignant pleural mesothelioma (MPM) is 20%, in contrast to 8% for distant MPM [11]. However, these data likely reflect cases involving larger tumors in which the patient undergoes larger, more invasive surgeries. The final unusual piece of data, in this case, is the lack of any evidence of prior exposure to asbestos.

## Conclusions

This case highlights the importance of LDCT scanning for early diagnosis of life-threatening illnesses, based on USPSTF guidelines. Also emphasized is the clinical decision-making that prompted the patient’s caretakers to persist in securing a diagnosis based on her risk factors. Given the rare nature of sarcomatoid mesothelioma, we hope the information provided can add to the few other case reports for a better understanding of this cancer.

## References

[1] Litzky LA (2020). Pathology of malignant pleural mesothelioma. UpToDate.

[2] (2022). Mesothelioma recent trends in SEER age-adjusted incidence rates, 2000-2019. https://seer.cancer.gov/statistics-network/explorer/application.html.

[3] Bridda A, Padoan I, Mencarelli R, Frego M (2007). Peritoneal mesothelioma: a review. MedGenMed.

[4] Bianco A, Valente T, De Rimini ML, Sica G, Fiorelli A (2018). Clinical diagnosis of malignant pleural mesothelioma. J Thorac Dis.

[5] Lanphear BP, Buncher CR (1992). Latent period for malignant mesothelioma of occupational origin. J Occup Med.

[6] Aberle DR, Adams AM, Berg CD (2011). Reduced lung-cancer mortality with low-dose computed tomographic screening. N Engl J Med.

[7] (2021). Malignant pleural mesothelioma. https://www.nccn.org/guidelines/guidelines-detail?category=1&id=1442.

[8] Baas P, Scherpereel A, Nowak AK (2021). First-line nivolumab plus ipilimumab in unresectable malignant pleural mesothelioma (CheckMate 743): a multicentre, randomised, open-label, phase 3 trial. Lancet.

[9] Ceresoli GL, Aerts JG, Dziadziuszko R (2019). Tumour Treating Fields in combination with pemetrexed and cisplatin or carboplatin as first-line treatment for unresectable malignant pleural mesothelioma (STELLAR): a multicentre, single-arm phase 2 trial. Lancet.

[10] (2022). Ph 2/3 study in subjects with MPM to assess ADI-PEG 20 with pemetrexed and cisplatin (ATOMIC). https://clinicaltrials.gov/ct2/show/NCT02709512.

[11] (2022). Survival rates for mesothelioma. https://www.cancer.org/cancer/malignant-mesothelioma/detection-diagnosis-staging/survival-statistics.html.

